# Digital Storytelling Review in a Pharmacy Self-Care Course

**DOI:** 10.3390/pharmacy10020045

**Published:** 2022-04-15

**Authors:** Jenna M. Mills, Jason W. Guy, Julie H. Oestreich

**Affiliations:** 1 Department of Pharmacy Practice, College of Pharmacy, University of Findlay, Findlay, OH 45840, USA; guyj@findlay.edu; 2 Department of Pharmaceutical Sciences, College of Pharmacy, University of Findlay, Findlay, OH 45840, USA; julie.oestreich@findlay.edu

**Keywords:** digital storytelling, pharmacy education, active learning, pharmacy, self-care

## Abstract

Digital storytelling is a type of active learning that allows instructors to simulate real-life situations through a series of connected videos. While this technique has been used in other healthcare education disciplines, its use in pharmacy has not been well documented. A digital storytelling model was incorporated in a required self-care pharmacy course to assess if the technique was helpful to improve the knowledge, confidence, and satisfaction of students. Due to a shift in online learning, the self-care course offered a remote exam review session containing a digital storytelling model, and this approach was compared to an in-person exam review that followed a lecture-based model held earlier in the course. Pre- and post-knowledge assessments were given to determine the impact of the digital storytelling review. There were 50 students involved in both sessions and there was a 70% response rate in the digital storytelling group and a 90% response rate in the lecture-based group. Students’ knowledge numerically improved, but not to a statistically significant level for most questions. Nonetheless, students reported more confidence (*p* < 0.05) in their ability to pass the upcoming exam following the digital storytelling review. Thematic analysis revealed that the digital storytelling session was engaging and interactive, though time-management and breakout rooms could be further optimized. Based on these results, exam review in a required self-care pharmacy course using a digital storytelling format may be a suitable method for students to apply course content and may particularly be of utility in online or hybrid courses.

## 1. Introduction

Digital storytelling is a specific type of active learning that combines narratives with technology and allows instructors to create a series of connected videos to present information on a particular topic [1,2,3]. While there can be several types of digital stories, such as informative or instructional narratives, personal accounts, or historical presentations [2,3], pharmacy education focuses on stories designed around healthcare topics. By integrating simulated patient information throughout a class session, the video series provides students with a narrative story, which has been shown in other disciplines to increase student reflection and active participation [4]. For example, one small descriptive study of occupational therapy students found that the digital storytelling model increased student reflection, especially for visual learners [5]. Another study reported that digital storytelling for nursing students was as engaging and fun as traditional methods, but also time-consuming [6]. Further, digital storytelling has been described to effectively portray illness from the patient’s point of view and as a beneficial addition to nursing students’ learning, as outcomes on questions related to the topics contained in the videos improved [7]. In the field of pharmacy, the immersive experience that digital storytelling provides can help simulate real-life patient scenarios for students to apply their knowledge and develop problem-solving skills. Despite this application, there is limited information describing how digital storytelling could be utilized in pharmacy education.

The Accreditation Council for Pharmacy Education (ACPE) sets the standards for College of Pharmacy curricula and requires the incorporation of active learning therein [8]. Generally, active learning can be integrated in the classroom in a variety of ways, such as team-based learning, patient simulations, discussion-based learning, and games [9,10]. Active learning improves students’ recall, engagement, attentiveness [11], and outcomes related to knowledge, including exam scores and course failure rates [12]. Despite these benefits and the potential to utilize digital storytelling in pharmacy education, its application as an active learning strategy in a self-care course has not been described.

Self-care represents a required didactic topic according to guidance from ACPE [8] and is defined as 'the independent act of preventing, diagnosing, and treating one’s illnesses with or without seeking professional advice’ [13]. In the pharmacy setting, self-care encompasses the management of self-treatable conditions and the use of nonprescription or over-the-counter (OTC) products [13]. Compared to other healthcare professionals, pharmacists possess extensive knowledge of OTC medications [14], and almost 90% of Americans live within 5 miles of a community pharmacy [15,16]. Thus, pharmacists are readily accessible to patients in their community and uniquely suited to assist with self-care needs [13]. Therefore, it is critical for students in Colleges of Pharmacy to develop skills and retain information related to self-care so that they can provide accurate recommendations for patients seeking advice [17]. The utilization of active learning strategies specifically in self-care instruction is recommended [18], and employing digital storytelling in this manner to engage students may enhance the retention of information compared to traditional methods.

In addition, many adjustments in higher education have increased the need for remote teaching. For example, many institutions recently addressed the challenge of simultaneously teaching students face-to-face in the classroom and remotely on a video platform during the COVID-19 pandemic. While this hybrid learning model complicated course delivery, it also provided opportunities for innovation. In this study, the digital storytelling model was applied out of the need to engage students during a time of online course delivery. There is potential for the digital storytelling format to be applied in person or online, however, considering the ability to apply the technology in either setting.

In light of the lack of data on the impact of digital storytelling on pharmacy students’ learning, this study sought to evaluate if digital storytelling could be a viable active learning strategy in pharmacy curricula. The objective of this study was to evaluate the effectiveness of a digital storytelling exam review session for improving students’ knowledge, confidence, and satisfaction compared to a lecture-based review in a self-care pharmacy course. We hypothesized that reviewing key self-care concepts utilizing the digital storytelling method would improve these elements. This research study is anchored in constructivist theory, as the investigators encouraged active, hands-on, collaborative, and experiential learning. By employing the digital storytelling format, students faced and responded to simulated patient care scenarios, allowing students to achieve a higher level of cognitive engagement [19]. In addition, the digital storytelling model implemented in this study utilized a spiral design, as students built upon their knowledge from the self-care course and pharmacy curriculum [19]. The instructional design of the digital storytelling format focused on key principles described by Bruner, which include providing context, utilizing a structured approach, and obtaining a deeper level of understanding compared to traditional lecture-based concepts [19]. To compare strategies, the lecture-based session was provided in person, while the digital storytelling review session was conducted using a video platform after a shift to online course delivery in the Fall of 2020.

## 2. Materials and Methods

This study was approved by the University of Findlay Institutional Review Board (study #1485). A midterm and final exam review session were provided to first professional year students in a required self-care pharmacy course at the University of Findlay. The midterm review session was provided in person and followed traditional lecture-based pedagogical delivery. The final review session was held remotely and utilized an innovative digital storytelling delivery. Both sessions were provided in the same semester with the same students. The traditional review prepared students for a midterm exam and the digital storytelling method provided a review for the final exam. Each review session was designed for a 50-min class period. Topics that were addressed during the traditional review included gastrointestinal disorders (nausea, vomiting, diarrhea, heartburn, intestinal gas, constipation, and anorectal disorders), pain, fever, insomnia, drowsiness, and fatigue. The digital storytelling review covered cough, colds, allergies, pain, fever, and ophthalmic, otic, and oral disorders. The midterm and final exam possessed the same assessment format and structure, including a closed-book policy as students did not have access to resources during the assessment.

To create the digital storytelling review model, instructors first developed patient case scenarios focused on key concepts in the course. These scenarios were then fashioned into scripts to depict small stories about patients presenting to the pharmacy for guidance related to their health. Applying details from the ‘patient’s story,’ the goal of the activity was to transform each simulated patient interaction into an immersive and connected story to ultimately emulate a real-life situation likely to be encountered in a community pharmacy. These patient case scenarios were recorded as videos in advance and then played during the final exam review session using a video conferencing platform. The interactive session was instructor-led; one video would be played, and then at least one question would be posed to students to apply their knowledge of the content. The checkpoint questions assessed various course objectives, including patient assessment, exclusion criteria, OTC product selection, nonpharmacologic therapies, and patient counseling. After each breakpoint in the story, students were assigned into breakout rooms to formulate an answer as a small group and encourage discussion, which was especially important in light of the remote nature of the review. The instructor would then invite students back to the main room to share their answers using polling or chat features and debrief on the segment before moving onto the next video and checkpoint question. This process was repeated until all videos in the scenario were played and breakpoint questions reviewed, marking the end of the ‘patient’s story.’

The investigators collected student feedback via post-surveys, which evaluated perceived confidence and satisfaction of the two different review styles (traditional versus digital storytelling). To compare, the investigators utilized a post-survey for both the traditional and digital storytelling method, which was emailed to students for voluntary completion after each respective review session and due the following day. Students had not attempted the exam before completing the post-survey. The post-surveys were adapted from research tools [7,20,21] and further developed using input from several faculty members at our institution. Both post-surveys contained the same 8 questions; the majority measured students’ perceptions of the review method using Likert-scale responses, while one question was open-ended for students to comment on strengths, weaknesses, or opportunities to improve the exam review. The surveys were administered using Google^TM^ forms, and no identifiable information was collected from students.

An additional knowledge assessment evaluated students’ content knowledge before and after the digital storytelling review. This separate pre- and post-assessment contained 6 multiple choice questions that focused on topics covered during the review session, such as allergic conjunctivitis, post-nasal drip cough, nonprescription analgesics, drug classes, and acetaminophen dosage. The knowledge assessment was not given with the traditional review, as different topics were addressed with the different review styles. Of note, no new content was taught during the review sessions as these were preparation sessions for an upcoming exam.

Investigators used the Mann–Whitney U and chi-square tests to analyze data with the alpha set at 0.05. A modified thematic analysis [22,23] compared open-ended analyses of both the traditional and digital storytelling review session models. A total of 3 reviewers categorized all student comments and then came to an agreement on the final categories for the analysis based on their individual reviews.

## 3. Results

Of the 50 students who participated in the course, 90% of the students completed the lecture-based review survey and 70% of students completed the digital storytelling review survey.

The results and comparison of the traditional and digital storytelling review session post-surveys are shown in Table 1. Of note, students felt more confident in their ability to pass the upcoming exam after the digital storytelling review compared to the lecture-based review (*p* = 0.009). Numerically, students also felt more prepared for the upcoming exam (*p* = 0.085), although this improvement did not achieve statistical significance. Students varied in their perceptions of how well they would do on the exam. Here, 5 students expected to receive an A, 11 students expected a B, 15 students expected a C, 4 students expected a D, and 0 students expected an F. Anticipated performance aligned relatively well with actual performance on the final exam (13 students received an A, 18 students received a B, 10 students received a C, 7 students received a D, and 1 student received an F). 

Students’ knowledge of select self-care topics was assessed before and after completing the digital storytelling review. Numerical improvement occurred for each of the six assessment questions. However, only one of the questions reached statistical significance (*p* < 0.001). See Table 2 for individual assessment question details and a comparison of the results. 

Table 3 and Table 4 highlight the most common themes, positive and negative, of the lecture-based and digital storytelling review sessions identified through thematic analysis.

Post-surveys overwhelmingly revealed that the lecture-based review session felt rushed to students (Table 3). On the other hand, the most common themes identified in the digital storytelling post-survey were enhanced engagement and interactivity, though time-management and breakout rooms could be further optimized (Table 4). 

## 4. Discussion

The results of this study showed that students preferred the digital storytelling design of the exam review session in the self-care course. Students also had increased confidence in their ability to pass their upcoming exam after completing the digital storytelling session compared to the lecture-based session. However, knowledge and exam readiness improvements were not compelling. Student scores improved after the digital storytelling session, but many of the results were not statistically significant. 

The major finding from this study is that a small subset of students found the digital storytelling design to be engaging and interactive, which can be of particular utility with online course delivery. Many courses have shifted to online delivery, and even if classes are conducted in person, students may temporarily require remote learning due to health or other personal reasons. This has presented challenges for instructors to effectively engage students in multiple modalities. This study offers one alternative approach to engage students who are participating in the class from a remote location. The study showed nonsignificant improvements in exam readiness and anticipation of a passing grade. However, students felt prepared and anticipated they would pass, which is encouraging as remote exam reviews were not the norm in the course and the final exam covered more content than a midterm exam did. One area that showed promise was that more students in the study felt engaged during the digital storytelling format compared to the traditional format. This suggests that even without major knowledge improvements, the pedagogical benefits and classroom atmosphere may warrant consideration of the extra time to design a digital storytelling session. Interactive approaches to content review could become increasingly impactful as teaching students simultaneously in a classroom and on a video platform has become more commonplace, and as classes are increasingly being offered online. Furthermore, innovative methods to connect with students participating remotely may enhance class rapport with faculty and other students.

One of the major features of a digital storytelling design is that it is easily transferrable to other disciplines and topics. The design of the session only requires creation of a case-based or original story related to the topic of interest. This flexibility means that all types of classes and topics could benefit from a digital storytelling design to improve engagement with content. In particular, the digital storytelling design could be especially impactful for topics that typically demonstrate low engagement or are considered uninteresting by students.

At 2 credit hours, the self-care course covers a range of topics, such as dietary supplements, fungal infections, cough, cold, allergy, nausea and vomiting, heartburn, and constipation. For each condition, students learn typical presentations and how to differentiate clinical features from other conditions, the appropriateness of self-care treatment, evidence-based management of both nonpharmacologic and pharmacologic treatments, key drug information related to pharmacologic strategies, and monitoring parameters. In general, instructors provide a lot of detailed content via traditional lecture, and each condition is usually covered in a stepwise manner during class. For example, students may first learn about clinical presentation, followed by exclusion criteria, and then nonpharmacologic and pharmacologic treatment strategies (where product-specific and safety considerations are covered) before ending with monitoring and follow-up information. The digital storytelling format could also follow a stepwise manner, as a series of connected short videos were played to portray a progressive patient case focused on one self-care condition. The videos built upon each other as more information was shared from the ‘patient’s story,’ as did the checkpoint questions (for example, students first determined if self-treatment was appropriate, then identified the most appropriate pharmacologic product, and lastly formulated patient counseling points).

Another benefit of the digital storytelling format is the ability for students to work together to problem-solve. Further, the digital storytelling session provided students with more case-based exercises to apply content than was previously provided in the course. One of the common themes from the traditional lecture-based review session was that students wanted more engagement and interactivity. Digital storytelling captures the settings and emotions of individuals’ stories [1,24], and in our experience, added elements to each patient case scenario for students to process and interpret. Thus, digital storytelling supplies an excellent medium to depict real-life scenarios to challenge students to utilize the information that is presented while calling upon their preexisting knowledge to formulate responses. 

### 4.1. Advantages

There are several advantages of incorporating the digital storytelling design in the classroom, including student engagement—as discussed above. Additionally, faculty can prepare exercises prior to class, which allows for scenarios that are high quality and well controlled. The digital storytelling design could focus on one topic at a time or include several topics in one patient case. By portraying complex patient case scenarios, instructors could highlight multiple health conditions in patients that need management, which students will inevitably face during experiential opportunities, internships, or future careers as pharmacists. Digital storytelling provides an outlet for students to practice facing complex patient care situations in the classroom and may help to prevent compartmentalized thinking. The decision of what and how much to include in a ‘patient’s story’ will likely depend on the students’ prior coursework, but demonstrates the ability of digital storytelling to be customized for the class.

### 4.2. Challenges

The digital storytelling model does have drawbacks associated with it. There was a large time commitment involved with creating and preparing the digital storytelling exam review session. Faculty spent approximately 10 h creating the scenario scripts and keys, recording the videos, and preparing the material to debrief after each checkpoint. The material may maintain relevance for more than one year though. Thus, the potential to reuse videos and keys could significantly decrease the amount of time spent on preparing for the digital storytelling review in subsequent years. 

The digital storytelling model also introduced several technology elements, which introduced potential for more logistical issues. For example, breakout rooms worked well for most groups, but some students reported frustrations with members not communicating during the checkpoints. This created problems because students were assigned to the same group for the entire review session. If faculty had randomized students into different breakout rooms at each checkpoint as opposed to just once at the initial checkpoint, this issue could have been avoided. Thus, based on our experiences, we recommend testing all features of the video platform system and orienting students prior to class to ensure a smooth class session. 

Finally, class time became an issue with the digital storytelling model. Each class session in the self-care course is limited to 50 min. The instructors attempted to review three different topics utilizing three different simulated patient cases/stories, which was difficult to complete in 50 min. Making sure that the digital storytelling session will fit in the allotted class time is an important consideration for faculty looking to utilize the digital storytelling model. Even if a timeline for the session is prepared in advance, the time spent on discussion with students after each checkpoint can be difficult to predict and may not fit into the time estimates. Thus, future installments could address time management issues by limiting the length and number of videos, as this may allow for a more streamlined class session. 

### 4.3. Limitations

This study focused on the impact of a digital storytelling session on students’ knowledge, confidence, and satisfaction using review material, not new material. As students had already learned the concepts earlier in the course, this might explain why dramatic improvements in knowledge were not observed after the digital storytelling review compared to before. Perhaps utilizing digital storytelling to teach new concepts may produce larger improvements in knowledge. Moreover, some of the videos could have been more engaging than others due to the creativity of the case or acting, which might explain why one topic scored significantly better on the post-knowledge assessment for the digital storytelling method compared to other topics. In the future, it will be important to optimize and streamline the videos and technology used in the digital storytelling review. 

In addition, while assessing students in the same cohort during the same semester helped to minimize the inter-student variability of results, the study had a modest sample size (*n* = 45) and was conducted in one course in one College of Pharmacy, which may limit results. Lastly, the investigators collected student feedback using post-surveys, which only measured students’ perceptions, consisted of a limited number of questions, and captured information in the moment of completion. Future studies could consider a more comprehensive evaluation of learning and assess higher-level skills and abilities, such as students’ problem-solving, creative thought processing, or communication [25,26] before and after a digital storytelling session.

## 5. Conclusions

In a pharmacy self-care course, we utilized a digital storytelling model as a method of exam review to immerse students in patient scenarios leveraging a series of connected videos and checkpoint questions. By simulating patient care scenarios, students reviewed topics previously taught in the course and responded to the actor’s problem or question, calling upon their knowledge of the topic at hand, and formulating a reply at each stage of the patient’s story. Following the digital storytelling review, students felt more confident in their ability to pass their upcoming exam. While knowledge scores remained similar between the traditional lecture-based and digital storytelling review designs, students described the digital storytelling model as engaging and interactive. Digital storytelling may be a useful method for students to apply course content and serve as a helpful tool when instructing students in a hybrid or remote course.

## Figures and Tables

**Table 1:** Post-survey traditional lecture vs. post-survey digital storytelling.

Topic Assessed	Mean Score Traditional Lecture	Mean Score Digital Storytelling	*p*-Value
Perceived readiness for upcoming self-care exam ^a^	3.02	3.34	0.085
Level of confidence in ability to pass upcoming self-care exam ^a^	3.53	4.11	0.009
Which exam review format do you prefer to increase understanding of self-care concepts? ^b^	2.87	3.29	0.222
Which exam review format is the most effective use of class time? ^b^	2.64	2.94	0.484
This exam review style enhanced my learning experience and engaged me ^c^	3.47	3.69	0.401
I would recommend this style of exam review to other students ^c^	3.47	3.66	0.453
What exam grade do you anticipate earning?	84% would pass	89% would pass	0.220

^a^ Likert-scale response: 1 = strongest negative response; 5 = strongest positive response; 3 = neutral. ^b^ Likert-scale response: 1 = strongest agreement with traditional; 5 = strongest agreement with interactive; 3 = neutral. ^c^ Likert-scale response: 1 = strongly disagree; 5 = strongly agree; 3 = neutral.

**Table 2:** Results of assessment questions before and after digital storytelling review.

Question Topic	Pre-Digital Storytelling Review Score Correct	Post-Digital Storytelling Review Score Correct	*p*-Value
Pharmacologic treatment for allergic conjunctivitis	66.7%	77.1%	0.327
Nonpharmacologic treatment for allergic conjunctivitis	22.2%	28.6%	0.539
Nonprescription drug recommended for post-nasal drip cough	58.3%	94.3%	< 0.001
Correct match of nonprescription drug and drug class	75%	88.6%	0.140
Nonprescription analgesic to avoid in patients with history of heart attack or stroke	88.9%	94.3%	0.414
Recommended dose of acetaminophen for pediatric patient	97.2%	100%	N/A

**Table 3:** Post-survey themes of traditional lecture.

Theme	*n* (% Responses)
Rushed	20 (44.4%)
Make more engaging/interactive	7 (15.6%)
Overall beneficial	4 (8.9%)
Content overwhelming	3 (6.7%)

**Table 4:** Post-survey themes of digital storytelling.

Theme	*n* (% Responses)
Overall beneficial	9 (25.7%)
Time not optimized	7 (20%)
Breakout rooms not effective	6 (17.1%)
Enjoyed the interactive format	3 (8.6%)

## Data Availability

Data is available in Table 1, Table 2, Table 3 and Table 4. Individual reported results can be made available by request to the corresponding author.
